# Implications of COVID-19 for nutrition

**DOI:** 10.1017/S136898002000436X

**Published:** 2020-12

**Authors:** Allison Hodge

With global case numbers over 40 million and more than one million deaths^(1)^ (as of 21 October 2020), the world eagerly awaits a vaccine against SARS-CoV-2. At PHN, we have fast-tracked corona virus articles and, in this issue, we publish four commentaries addressing potential impacts of the pandemic on aspects of nutrition. From Brazil, Pereira and Oliveira^(2)^ discuss poverty and food insecurity. Parekh and Deierlein^(3)^ from the USA note the potential for changes in health behaviours related to public health responses to the virus that may promote obesity and related chronic diseases, all of which increase the risk of suffering severe illness with corona virus infection. Both Jacob etal^(4)^ and Attwood and Hajat^(5)^ discuss the risks of zoonoses, such as that caused by SARS-CoV-2, associated with meat consumption and the implications of this.

In response to COVID-19, many countries have imposed restrictions on leaving home and closed businesses which is a logical approach to reducing infection but may have other consequences. In the USA, a country where around 42 % of the population are obese, which increases the risk of adverse consequences of COVID-19, Parekh and Deierlein^(3)^ argue that lockdown could promote behaviours contributing to obesity. Looking at sleep, physical activity/sedentary time, diet and alcohol intake, they discuss how stress and job insecurity could lead to poor sleep and alcohol consumption; availably, cost of and access to fresh food and the trend towards storing shelf-stable foods may reduce diet quality, and being restricted to home could reduce physical activity and increase time spent in screen-based activities. Individual-level advice is provided addressing each of these behaviours but can only work when supported by community- and policy-level interventions that ensure access to appropriate foods and facilities for physical activity, for example.

Especially in poorer countries, responses to COVID-19 are likely to increase poverty, and food and nutrition insecurity^(2)^. People who are unable to work because of COVID-19 may not be able to afford food, but also changes to food production and transport may limit availability, making it even more difficult to consume an adequate diet. Good nutrition is important for a healthy immune system, so food and nutrition insecurity may increase vulnerability to infection with SARS-CoV-2 and to more severe consequences of infection^(6)^. This article emphasises the need for governments at all levels to intervene and ensure that all citizens are free from food and nutrition insecurity.

Food and nutrition insecurity may also be an issue for some populations if suggested bans on consuming wildlife come into place. It is understood that SARS-CoV-2 and other important infections have spread into humans from wild animals and banning such consumption has been debated as a response to reduce the risk of future zoonoses. Jacob etal^(4)^ argue that this could have unintended consequences for people who do not have other sources of dietary protein or rely on selling bushmeat for income. Hunting and consuming wildlife are also part of the cultural heritage of some communities which would also need to be considered if the practice was to be banned. These authors also argue that alternative sources of animal foods, such as intensive agriculture, are not without risks. Well-known epidemics related to animal agriculture include H5N1 avian flu and Nipah virus in pigs. Hence, the conclusion that all animal-based food systems offer health threats, and we should take the opportunity to consider reducing our level of meat consumption, with benefits to both human and environmental health.

Attwood and Hajat^(5)^ also point to previous zoonoses reducing intake and changing choices of meat. Not only are animals now understood to be sources of infection but also meat processing plants have been identified as the sites of COVID-19 outbreaks globally^(7)^. As people are not eating in restaurants during COVID-19, they may be consuming less meat and long-life staples that some have stocked up on do not include meat. These changes have contributed to recent trends in wealthier countries where meat intakes have tended to decrease. For example, the National Diet and Nutrition Survey 2008/2009–2016/2007^(8)^ in England found a downward trend in the intake of red and processed meat over the study period. Whether these changes are maintained in the long term is yet to be seen but given that COVID-19 is likely to be with us for a while, yet the impacts may be longer lasting.

What these commentaries highlight is that the response of governments to the corona virus pandemic needs to include more than just testing for the virus and treating infected people. The long-term outcomes of COVID-19 may include changes in what people choose to eat, away from animal-based foods, which has potential benefits for human and planetary health. Any such change needs to account for those who do not have the option to choose.
